# Targeting the link between late pregnancy and breast cancer

**DOI:** 10.7554/eLife.01926

**Published:** 2013-12-31

**Authors:** Balabhadrapatruni VSK Chakravarthi, Sooryanarayana Varambally

**Affiliations:** 1 **Balabhadrapatruni VSK Chakravarthi** is in the Michigan Center for Translational Pathology, Department of Pathology, University of Michigan, Ann Arbor, United States; 2 **Sooryanarayana Varambally** is in the Michigan Center for Translational Pathology, Department of Pathology and the Comprehensive Cancer Center, University of Michigan, Ann Arbor, United Statessoory@med.umich.edu

**Keywords:** STAT5, pregnancy, breast cancer, chemoprevention, carcinogenesis, Human, Mouse

## Abstract

Why does a first pregnancy after age 35 increase the risk of breast cancer, and what can be done to combat this?

**Related research article** Haricharan S, Dong J, Hein S, Reddy JP, Du Z, Toneff M, Holloway K, Hilsenbeck SG, Huang S, Atkinson R, Woodward W, Jindal S, Borges VF, Gutierrez C, Zhang H, Schedin PJ, Osborne CK, Tweardy DJ, Li Y. 2013. Mechanism and preclinical prevention of increased breast cancer risk caused by pregnancy. *eLife* **2**:e00996. doi: 10.7554/eLife.00996

**Image** Pregnancy can accelerate the development of precancerous breast cells into cancerous lesions

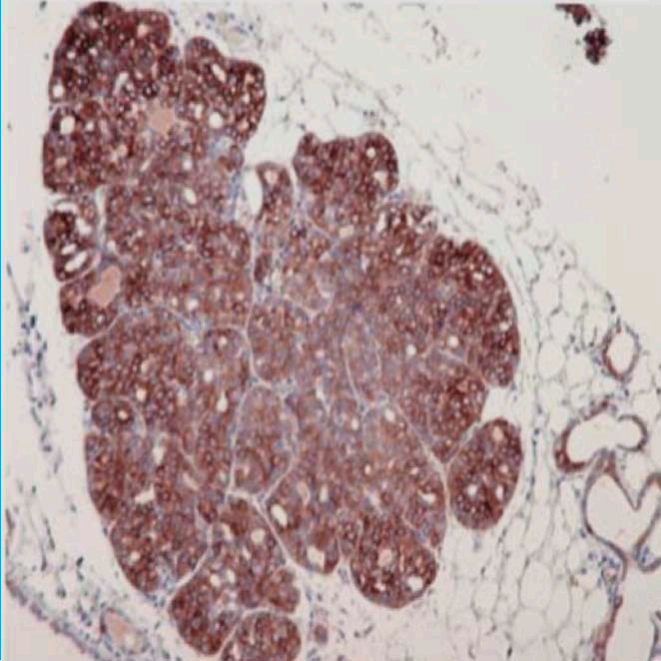

Breast cancer is the leading cause of cancer deaths in women, killing more than 40,000 women in the US alone last year (American Cancer Society, 2013). It is also a highly variable disease, and the significant levels of diversity between different tumours, or within the same tumour, make the diagnosis and treatment of breast cancer a challenge. Pregnancy is one factor that is known to influence the chances of a woman developing breast cancer. Women who become pregnant and have children at an early age have a decreased risk of developing breast cancer in later life; however, any pregnancy after age 35 increases the risk of breast cancer (Polyak, 2006).

Pregnancy causes extensive changes to the breasts, making breast cells less likely to multiply, and also less likely to develop tumours—which could explain the protective effect of pregnancy for younger women. However, it is unclear why becoming a first-time mother at an older age has the opposite effect. After age 35, breast tissue is more likely to have accumulated cells carrying cancer-causing mutations, or clusters of abnormal cells with the potential to become cancerous: however, it was not known how these cells were affected by a late-age first pregnancy. Now, in *eLife*, researchers at Baylor College of Medicine, the MD Anderson Cancer Center and the University of Colorado in Denver report that the answer to this question lies in a signalling pathway called the JAK-STAT5 pathway (Haricharan et al., 2013).

The mouse models that are conventionally used to study cancer have been genetically engineered to carry cancer-causing genes, or oncogenes, that are expressed in response to the same hormones that control pregnancy and milk-production. As such, these models are not ideal to study the effect of pregnancy on the development of cancer. Additionally, since transgenic oncogenes will often impair the normal development of the mammary gland, it is particularly difficult to address the effect of pregnancy on pre-existing breast tissue cells using these traditional mouse models.

However, injecting an engineered virus that encodes an oncogene into the nipples of mice circumvents these issues. The resultant viral infection results in these oncogenes being expressed in cells within a normally developed mammary gland (Du et al., 2006). Now, Yi Li of Baylor and colleagues—including Svasti Haricharan and Jie Dong as joint first authors—have used this technique to investigate the effect of pregnancy on pre-existing precancerous cells to see if this can explain the increased risk of developing breast cancer seen in older first-time mothers.

Haricharan, Dong et al. introduced two cancer-causing genes that are frequently altered in human breast cancers, *ErbB2* or *Wnt1,* into female mice. Later, once precancerous cells had already started to develop, half of the mice were made pregnant and allowed to wean their young, whilst the other half remained virgins. Both genes eventually induced tumours in both sets of mice, but more tumours developed in the mice that had been pregnant than in the control mice, and they also developed much more rapidly. Most of the tumours initiated by ErbB2 in the mated mice appeared at least seven weeks after the completion of pregnancy, which is too late to be considered as a case of pregnancy-associated breast cancer: in humans, breast cancer diagnosed during pregnancy, breast-feeding, or within one year of giving birth is called pregnancy-associated breast cancer (Borges and Schedin, 2012). Wnt1-induced tumours appeared more than 36 weeks after the completion of pregnancy, again ruling out pregnancy-associated breast cancer as the cause of the cancer.

Pregnancy accelerated the development of oncogene-activated, precancerous cells into cancerous lesions and tumours. In the mice, at time points when the young were still suckling, and during involution (the period after birth when the womb shrinks back to its pre-pregnancy size), the mated mice had more early lesions than age-matched unmated mice. However, the rate of cell proliferation was equal in both groups, which suggests that this phenomenon is not due to pregnancy causing precancerous cells in the mated mice to divide more frequently than in the control mice. Instead, the early lesions of the mated mice showed fewer signs of the programmed-cell death responses that would eliminate abnormal or precancerous cells that were seen in the unmated mice. Staining for cleaved caspase 3—a tell-tale sign of programmed cell death, or ‘apoptosis’—showed that pregnancy allows the precancerous breast tissue cells to escape processes that would normally cull abnormal cells. Haricharan, Dong et al. show that this occurs because precancerous cells in the pregnant mice activated the pro-survival machinery inside the cells to counteract the pro-cell death responses.

The activated form of the protein STAT5, called pSTAT5—which has pro-survival activity—is detectable in most epithelial mammary cells, and its levels increase during pregnancy and lactation. The pregnancy hormones, placental lactogen and prolactin, are recognized by the receptor called PRLR, which causes the enzyme Jak2 to activate STAT5 and converting it to pSTAT5 (Hennighausen and Robinson, 1998). Haricharan, Dong et al. showed that—unlike their normal counterparts—oncogene-activated mammary cells continue to maintain high levels of pSTAT5 and fail to degrade PRLR during involution. Moreover, mammary epithelial cells harbouring degradation resistant PRLR display oncogenic properties (Plotnikov et al., 2009). These data suggest that during pregnancy and lactation, pre-existing precancerous mammary cells activate the PRLR-Jak2-STAT5 signalling cascade (Figure 1) and aberrantly maintain this activated state long after the end of pregnancy, which increases the survival of pre-cancerous cells and hastens cancer progression.

**Figure 1.**
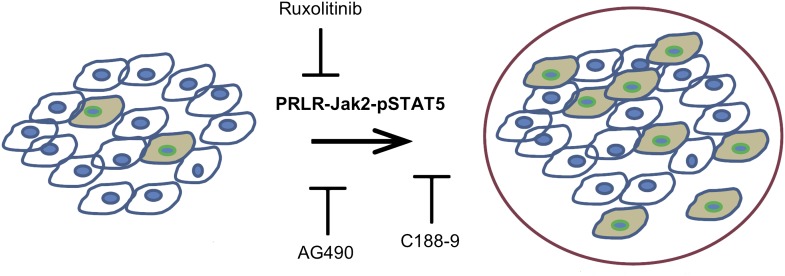
Blocking Jak2-STAT5 activity can reduce the breast cancer risk associated with late-age pregnancy. During pregnancy (left), pre-existing precancerous cells (filled in) activate the PRLR-Jak2-STAT5 signalling pathway, accelerating their progression to fully cancerous cells (right). Haricharan et al. show that this pathway can be blocked by various molecules, including Ruxolitinib, AG490 and C188-9.

Using AG490, an inhibitor of JAK-STAT signalling that blocks several tyrosine kinases including Jak2 (Gazit et al., 1991) as well as the more specific JAK kinase inhibitor, Ruxolitinib (Quintas-Cardama et al., 2010), Haricharan, Dong et al. show that Jak2 signalling is required for early lesion survival and progression in the mated mice. Furthermore, C188-9, an inhibitor that blocks STAT5 activity (Redell et al., 2011) caused a significant decrease of pSTAT5 positive cells, increased apoptosis, and resulted in a dramatic regression of premalignant lesions specifically in the mated mice. These results suggest that targeting STAT5 activity may lower breast cancer risk in women who have had a late-age pregnancy as well as in those who have abnormally high levels of pSTAT5.

The work of Haricharan, Dong et al. could lead to more effective strategies to both treat breast cancer and to reduce the incidence of breast cancer. Moving forward, validation of the present study and additional pre-clinical trials of STAT5 pathway inhibitors might pave the way for clinical trials in humans to reduce the risk of late-age pregnancy associated breast cancer risk.
